# Efficiency, Efficacy, and Power in the Implementation of a Medication Adherence Aid

**DOI:** 10.3928/24748307-20180525-01

**Published:** 2018-07-11

**Authors:** Roy Cherian, Urmimala Sarkar, Elaine C. Khoong, Sara Ackerman, Gato Gourley, Dean Schillinger

## Abstract

Nonadherence to medication regimens is common, with approximately 50% of patients not taking their medications as prescribed. The Universal Medication Schedule (UMS) is a set of standardized, evidence-based, and patient-centered instructions for pill-form medications that has demonstrated improvements in adherence by promoting patient comprehension. An urban, publicly funded, integrated health care system attempted to adopt UMS labeling but had limited success at its largest pilot site, which was a safety-net health care system's outpatient pharmacy. To assess barriers to implementation, we engaged pharmacists at this site in group interviews. We thematically analyzed transcripts by integrating sociological work on standardization with grounded theory methodologies. In addition to lacking technological infrastructure, tensions among efficiency, efficacy, and effectiveness, and tension between individual/biomedical versus population health perspectives emerged as barriers to implementation. Additionally, we discovered that hierarchies of professional power impeded uptake. For successful implementation of evidence-based practices for vulnerable populations in resource-poor settings, efforts must anticipate and reconcile the tensions among conflicting demands, professional hierarchies, and divergent orientations to patient care. **[*HLRP: Health Literacy Research and Practice*. 2018;2(3):e128–e131.]**

Medication nonadherence is common, with approximately 50% of patients not taking their medications as prescribed (Sabate, 2003). Nonadherence not only diminishes the potential efficacy of treatment strategies, but can also contribute to adverse drug events (Bailey et al., 2011; Budnitz, Lovegrove, Shehab, & Richards, 2011; Steinman, Handler, Gurwitz, Schiff, & Covinsky, 2011). One cause of nonadherence is difficulty interpreting medication instructions (Bailey et al., 2011). Traditional medication instructions often omit specifications regarding timing, indication, and frequency (Tarn, Paterniti, Orosz, Tseng, & Wenger, 2013; Wolf et al., 2009) while employing technical terms (e.g., “subcutaneously”) that can cause confusion among patients with limited health literacy (Bailey et al., 2014; Davis et al., 2006; Fang, Machtinger, Wang, & Schillinger, 2006). Additionally, medication instructions are not standardized; a study showed that prescribers have 53 different ways of conveying “take 1 tablet a day” on pill bottles (Bailey, Persell, Jacobson, Parker, & Wolf, 2009).

One evidence-based approach to enhance the comprehensibility of prescription instruction is the Universal Medication Schedule (UMS) (Wolf et al., 2009), a set of standardized, plain-language instructions (National Council for Prescription Drug Programs, 2013). Emerging evidence suggests that better comprehension translates into improved medication adherence and clinical outcomes (Wolf et al., 2011). Although UMS improves comprehension rates across literacy levels, it most strongly benefits those with low literacy, limited English proficiency, and complex medication regimens (Wolf et al., 2011).

In response to a regulation passed by California's State Board of Pharmacy (2012) requiring pharmacies to dispense medications with patient-centered labeling, the San Francisco Health Network, an urban public delivery system, began an initiative in November 2015 requiring its publicly owned and operated pharmacies to dispense medications with UMS instructions (Wolf et al., 2011). The Ambulatory Safety CEnter for INnovaTion (ASCENT), funded by a National Institutes of Health grant (P30HS023558-01), led this effort.

## Objective

This article describes barriers to UMS implementation and strategies to promote future uptake at the largest implementation site—the Zuckerberg San Francisco General Hospital outpatient pharmacy (OP)—where conversion rates were suboptimal (**Table 1**).

## Methods

### Study Setting

The OP prepares and processes an average of 310 prescriptions a day. Its staff is comprised of 10 pharmacists and 10 pharmacy technicians. Prescribers include 749 physicians and nurse practitioners caring for patients at on-site primary care clinics and specialty clinics, as well as at off-site community primary and urgent care clinics.

### Participants and Procedures

We conducted group interviews with front-line pharmacists in April 2016 to understand perceptions of the UMS and inform them of its ongoing implementation. The University of California San Francisco's Institutional Review Board approved this study. The research team provided the pharmacy supervisor with a flyer indicating the dates, times, and reason for the group interviews to share with pharmacists 1 week prior.

At the focus group, the first author read the consent form aloud and solicited any clarifying questions before obtaining verbal consent. The research team did not collect any demographic data or identifying information from participants. We asked participants to reflect on how technological infrastructure affected implementation and how pharmacists envisioned the UMS's impact on comprehension, adherence, and patient safety. The first group interview involved four clinical pharmacists and one pharmacy graduate student, and the second interview involved two clinical pharmacists and one pharmacy student. Each group interview lasted 1 hour. Interviews were audio-recorded and transcribed. The study team de-identified the transcripts to ensure anonymity.

### Analysis

The first author (R. C.) extracted excerpts from the transcripts using Dedoose 7.5.9. He then categorized excerpts into themes using a grounded theory approach by (1) writing memos for each excerpt to distill their content, (2) using words and phrases present in the excerpts to derive a coding scheme (Charmaz, 2006; Glaser, 1992), and (3) grouping these codes into an emergent theoretical framework.

## Key Results

### Conflicting Demands

In both group interviews, pharmacists remarked upon the difficulty of reconciling UMS conversion with their current workflow:

[W]e were just “Leaned” so they want efficiency. They want better turnaround time… Well in this department, we don't actually type the labels, the technicians do. So by the time we get the order, the technician's making a big jump in assumption about what the scheduling should look like. So if we get it and the technician decided that well, it should be taken in the morning and maybe it's something like Paxil which has a lot of side effects and stuff, and as you said, I exercise my clinical judgment. Well what happens? I mean, it's a hard stop. Because now I've got to take everything that's presented to me, I have to run it back, put it in the holding queue and have it redone and it [increases] the turnaround time.

Because the health system was unable to modify the electronic health record to automate UMS instructions at the point of prescribing, they instead relied on pharmacy technicians manually converting instructions at dispensing. However, technicians often lacked the clinical judgment required to make these conversions, resulting in redundancies in the workflow.

Clinical pharmacists pointed out the contradiction between the emphasis on efficiency related to reducing the time needed to dispense a prescribed medication and the time required for manual UMS conversion. Without an information technology system that could automatically convert instructions into the UMS in a way that aligned with clinical judgment, pharmacy staff felt they lacked the necessary resources to implement UMS efficiently and effectively.

### Professional Culture: Perceived Liability and Transgressing Authority

Institutional approval and mandates did not allay pharmacists' concerns surrounding the legality of conversion, which emerged most strongly with regard to transferring prescriptions to other pharmacies:

Say we transfer a prescription over to another pharmacy… Now, is Walgreens really getting the original of what the doctor prescribed? Not really. Because you prescribed three times a day and we're saying morning, lunch, and dinner. Is that really legal in a way?

The concern for medico-legal liability was intimately related to concerns of harming patients. Because UMS adoption is not yet widespread, pharmacists were concerned that UMS implementation at the OP might lead to patients receiving inconsistent instructions, causing confusion and ultimately harm:

Let's say the patient goes to multiple pharmacies, and they get the medication from us saying morning, noon, and night. The next prescription they bring it to MOMS [mail order medications] Pharmacy…and they just enter as what the doctor typed, three times a day. Now, the patient gets confused like, “Well, okay. How am I supposed to take this now? I have different directions.”

Experiential knowledge of patients who have been confused with UMS instructions that contradicted or differed from longstanding instructions heightened pharmacists' fear and anxiety that UMS may do more harm than good.

Despite institutional approval, professional hierarchies made pharmacists feel it necessary at times to consult with prescribing physicians before converting medication instructions into UMS. As with the concern for patient confusion and the risk for harm, the fear of transgressing physician's authority was not hypothetical:

One doctor called in a prescription for Lipitor, a statin, that under the UMS policy has to change everything to the evening, so I try to get the doctor on the phone to put it in the evening. I got yelled by the doctor saying, “Lipitor is one of the statins that doesn't need to be taken in the evening.”

### Standardization Versus Individualization

Drawing more from training in pharmacokinetic principles rather than the literature on UMS, pharmacists believed that traditional instructions will produce greater biochemical homeostasis due to more uniform intervals between administration than the intervals that are indicated in the UMS.

For us as pharmacists, when we go to school and say if a pill is supposed to be taken three times a day, and then you automatically are thinking about it should be spaced out every eight hours…because you studied the drug, how it is absorbed, or how you could [you know, metabolize] supposed ideally.

Furthermore, pharmacists implied that population-level standardization is not appropriate for vulnerable populations whose daily lives are not simple or predictable. Pharmacists claim that patients in the “safety-net” health system lack the autonomy to administer medications according to the UMS, which assumes normative, or at the very least consistent, definitions of morning, midday, and evening. In contrasting the safety-net population to a more affluent population, the pharmacist said:

The populations that we take care [of] here, with multiple comorbidities, lifestyles, and daily schedules, are so varied. My day could be somebody's night if they work nights…you have to be able to incorporate each individual's life schedule.

Pharmacists conveyed that interventions to improve adherence should focus on tailoring medication schedules that accommodate individual patients' structural vulnerabilities (e.g., food or housing insecurity). When acknowledging that physicians may not possess the requisite time to individually tailor instructions, pharmacists concluded that the variability and ambiguity of traditional instructions are actually more accommodating of the daily lives in vulnerable populations.

## Limitations

We were unable to speak with pharmacy technicians. Based on pharmacists' comments and the centrality of the pharmacy technician with respect to workflow, their exclusion likely means that we have an incomplete picture of the barriers of UMS implementation. Similarly, we were unable to assess or report on patient perspectives of the UMS. In addition, because our focus was on the lowest-performing and highest-volume site, this article does not examine the facilitators that likely were present at the three other sites. Lastly, we cannot generalize these findings given the nature of the sampling and qualitative research methodology.

## Conclusions

Due to the limited resources in the face of the demands for complex multidisciplinary care, health care in the safety-net system requires striking a balance among efficiency, efficacy, and population health. Doing so while integrating quality improvement initiatives within “leaned” workflows can be complex, especially when technological infrastructure is lacking. Concerning UMS implementation at one health system's OP, a lack of flexibility in professional roles, compounded by inadequate technological infrastructure, produced an environment lacking the agility to implement evidence-based practices in sites for which they were designed. With the exception of inadequate technological infrastructure, these group interviews revealed actionable barriers to application, such as step-wise implementation, active evaluation, and prescriber education and “buy-in.”

Since these interviews, implementation has become stepwise to give pharmacy technicians the time to learn optimal administration times for categories of drugs, thereby aligning conversion with clinical judgment and reducing redundancies. We are now conducting interviews with patients to assess how UMS instructions affect adherence and accuracy, which will shed light on pharmacists' skepticism about standardization. Lastly, after the group interviews, the Chief Medical Officer of the San Francisco Health Network sent a memo reminding prescribers of the conversion policy along with a survey to assess their perceptions of UMS. Although the response rate was low (29.8%, *n* = 223), support for UMS was overwhelmingly positive, with only 4.5% disagreeing with the conversion of traditional instructions into UMS.

Despite the evidence supporting the UMS and its potential impact on clinical outcomes for vulnerable populations, limitations in technological infrastructure and tensions in professional hierarchies as well as different definitions of and orientations to patient-centered care hindered local implementation. Future implementation efforts and research should actively evaluate UMS in local settings while being mindful of these potential barriers to promote uptake.

## Figures and Tables

**Table 1:** UMS Implementation Outcomes by Site

**SFDPH Pharmacy Sites**	**Pre-Implementation**	**Post-Implementation**
Behavioral health	0% (0/34)	94% (30/32)
Jail health	14% (11/80)	99% (85/86)
Skilled nursing facility	34% (54/158)	88% (541/612)
Outpatient pharmacy	23% (991/4,317)	23% (835/3,571)

Note. SFDPH = San Francisco Department of Public Health; UMS = Universal Medication Schedule.
